# Factors Contributing to Poor Self-Rated Health in Older Adults with Lower Income

**DOI:** 10.3390/healthcare9111515

**Published:** 2021-11-06

**Authors:** Mikyong Byun, Eunjung Kim, Heuijune Ahn

**Affiliations:** 1 BK21FOUR R&E Center for Learning Health Systems, College of Nursing, Korea University, Anam-dong, Seongbuk-Gu, Seoul 02841, Korea; mulanbb@korea.ac.kr (M.B.); ejissy@gmail.com (E.K.); 2 Department of Nursing Science, Catholic Kwandong University College of Medicine, 24 Beomil-ro 579beon-gil, Gangneung-si 25601, Gangwon-do, Korea; 3 Department of Hematology and Oncology, Gangneung Asan Hospital, University of Ulsan College of Medicine, 38 Bangdong-gil, Sacheon-myeon, Gangneung-si 25440, Gangwon-do, Korea

**Keywords:** self-rated health, risk factor, older adults, low income

## Abstract

Lower household income is associated with poorer self-reported health status, especially in the elderly. Considering the importance of subjective health in this fragile population, it would be worthwhile to explore the physical and mental health factors that may help to predict good or poor self-rated health (SRH) status. We first described three main categories (individual, physical, and psychological) between low-income seniors with good and poor SRH. Next, statistically significant physical and mental health factors affecting poor SRH were identified. In this study, original data from the 2017 National Survey of Older Persons in South Korea were analyzed. People aged 65 years and over with low household income were eligible. A total of 1405 men and 2945 women (*n* = 4350) were enrolled, and less than half of participants (47.5%, *n* = 2066) belonged to the poor SRH cohort. We applied individual variable-adjusted models and found that poor SRH was significantly associated with ADL limitation (odds ratio (OR): 2.91, 95% confidence interval (CI) 2.11–4.01), IADL limitation (OR: 1.80, 95% CI: 1.52–2.13), malnutrition (OR: 1.76, 95% CI: 1.53–2.04), and depression (OR: 3.65, 95% CI: 3.10–4.31) on logistic regression analysis. Our findings suggest that limited ADL/IADL, poor nutrition, and depression need to be emphasized to improve subjective health status in low-income adults. Early recognition and timely intervention might help them to live better and happier, ultimately relieving social healthcare burdens.

## 1. Introduction

In this era of worldwide aging, the rapidly increasing number of the aged population unavoidably results in personal, national, and global healthcare issues. As a matter of fact, the Republic of Korea is now one of the world’s fastest aging countries. Our country officially became an aged society in 2017 (which was just 17 years after we reached aging society status in 2000) and is estimated to enter a super-aged society by the end of 2026 [1]. Accordingly, the proportion of the elderly population has risen sharply, and this means that more and more people are exposed to health problems, economic difficulties, loneliness, and feelings of alienation as they get old. In 2020, our national health and welfare expenditure for this special population was KRW 12 trillion, which was equivalent to 1% of the gross domestic product (GDP) [2].

Another hot topic of the present time is the phenomenon of economic polarization resulting in health inequality among members of society. In other words, we live in a ‘looks equal but not equal’ community where individual health status depends on socioeconomic backgrounds such as education and household income despite the help from the social welfare system. When it comes to senior adults, this social problem gets even worse. It was found that people with low income levels have poorer functional/physical capability and worse psychological well-being [3].

Self-rated health (SRH) status is clinically important because this can serve as a predictor of one’s future morbidity or mortality, especially in the elderly [4,5,6,7,8]. Subjective health assessment is a relatively feasible and reliable tool to assess the responder’s overall health. It incorporates physical health factors, as well as mental factors such as overall sense of well-being and satisfaction in daily life [9,10]. A ‘poor or negative’ self-assessment of health represents not only physical distress but also emotional and social conditions of the individual. Thus, poor SRH seemed to be strongly associated with chronic illness, frequent hospital visits, and even mortality [11].

Considering the importance of SRH in this vulnerable population, it would be meaningful to explore the physical and mental health factors that may help to predict poor SRH in low-income adults. Using large-scale national data, we compared individual, physical, and mental health factors in those with good and poor subjective health groups. Then, we tried to identify significant physical and mental health variables affecting SRH by controlling for individual subcategories.

## 2. Materials and Methods

### 2.1. Source of Data

We obtained original data from the 2017 National Survey of Older Persons (NSOP) with approval from the National Statistical Office [12]. The database was constructed through random stratification of 10,299 Korean adults living in general residential facilities and was designed to represent the elderly population. The national survey was conducted from June to August 2017 through direct interviews with senior citizens aged 65 and over in 934 survey areas. Surveys were carried out by 60 professional surveyors (fifteen teams of four surveyors each, one supervisor in a team) who were previously trained by skilled research personnel.

### 2.2. Participants Selection and Study Design

**Figure 1:**
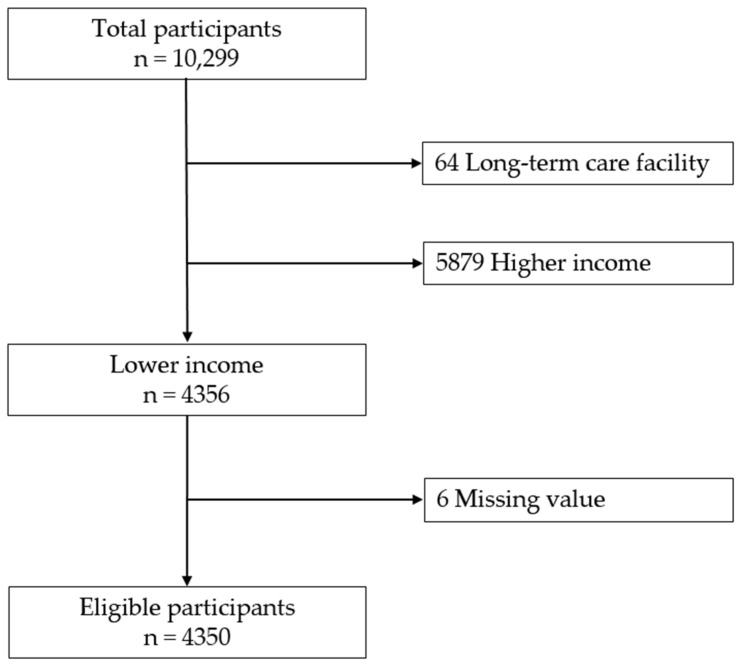
Flow diagram of inclusion of study population.

In this survey, household income was measured by totaling annual income from any (personal or public) sources. We divided our study population into five categorical income groups, called quintiles. The first quintile refers to those with the lowest income, and the fifth quintile the highest (i.e., Q1 (the lowest quintile), Q2, Q3, Q4, and Q5 (the highest quintile)). A person with ‘lower household income’ was defined as one who belonged to either Q1 or Q2. Older adults who met the following criteria were eligible: (a) lower household income, (b) community dwelling, and (c) without missing data. Finally, 4350 respondents were enrolled in this study (Figure 1).

Using descriptive and correlational study designs, we evaluated predictors of poor subjective health in adults with lower income with respect to individual, physical, and psychological categories. We then built variable-adjusted prediction models to identify physical and psychological factors that correlated with poor SRH in logistic regression analysis.

### 2.3. Measurements

#### 2.3.1. Subjective Health Assessment

Using a 5-point Likert question (“In general, how would you rate your current health status?”), we assessed the self-rated health status of the participants. Five answers were classified into dichotomous values: “good” (very good, good, and fair) and “poor” (poor and very poor).

#### 2.3.2. Individual Variables

Variables in individual categories were classified into four subcategories: demographic, socioeconomic, health status, and health-related behavior. Age, gender, marital status (living with a spouse or living without a spouse), and living status (alone, living with a spouse, living with children, and others) belonged to the demographic subcategory. Education level (0–6 years, 7–9 years, 10–12 years, or ≥13 years) belonged to the socioeconomic subcategory. Body mass index (BMI), chronic illness (such as arthritis, diabetes, and hypertension), and current number of medications (0, 1, 2, or ≥3) were categorized as the health status subgroup. Finally, the health-related behavior subgroup was made up of exercise, smoking (never/past or current), and drinking.

The presence or absence of chronic disease was evaluated by questions such as “have you been suffering from any diseases like high blood pressure, diabetes, or arthritis for more than 3 months?” and “have you ever been diagnosed with the above diseases by a doctor?”. Subjects who replied “yes” to both questions were determined to have a chronic disease. Physical exercise of 150 min or more per week was considered to correspond to the recommended level according to the World Health Organization (WHO) criteria [13]. The amount of exercise was classified as within the recommended level, below the recommended level, and none. Alcohol intake was evaluated on the basis of the National Institute on Alcohol Abuse and Alcoholism criteria [14]. Drinking less than one standard drink (a 350 mL glass of beer) per day was considered as an acceptable amount and drinking more than one standard drink a day was regarded as excessive in this study. The others who did not drink at all were determined as “none”.

#### 2.3.3. Physical Variables

Variables in the physical category encompassed visual, auditory, activities of daily living (ADL), instrumental activities of daily living (IADL), and nutritional status.

Visual discomfort was classified as “discomfort” in older adults who replied “uncomfortable” or “very uncomfortable.” Auditory discomfort was defined as “discomfort” in those who responded “uncomfortable” or “very uncomfortable.”

The ADL evaluation was performed in reference to the Korean Activities Daily Living scale [15]. This tool consists of seven queries: “bathing”, “bowel and bladder continence”, “dressing”, “eating food”, “face washing, brushing teeth, and shampooing”, “getting up and walking across the room”, and “toilet use”. Each query was estimated by a three-point rating (total independence, partial dependence, and total dependence), and a higher score indicated more severe limitations to daily routine. For example, a score of 0 (total independence) was regarded as “no limitation”. Scores 1 and 2 (partial and complete dependence, respectively) were regarded as “limitations”. Respondents who had ALD limitation in more than one query were considered to have “limitation to ADL”.

The presence of IADL limitation was estimated according to the Korean Instrumental Activity of Daily Living scale [15]. This scale is composed of ten domains: “ability to make and receive phone calls”, “going out for a short walk”, “laundry”, “managing money”, “performing household chores”, “personal grooming”, “preparing meals”, “shopping”, “taking medications on time”, and “using public transportation”. Total independence fell into “no limitation,” and the others (such as partial, complete, little, much dependence, and cannot be done at all) were recorded as “limitation”. Respondents with restrictions in more than one query were assessed to have “limitation to IADL”.

Individual nutritional status was evaluated on the basis of the ‘Determine Your Nutritional Health’ questionnaire from the Nutrition Screening Initiative [16], which consists of ten binary (“yes” or “no”) questions. A “yes” response to each item was scored in a range of 1 to 4, whereas a “no” response was scored as 0. The total score from 10 items was classified as good nutrition risk (0–2), moderate risk (3–5), and high risk (≥6). In our study, good nutrition risk was defined as “good nutrition”, and moderate and high risk were considered to be “poor nutrition”.

#### 2.3.4. Psychological Variables

Depressive mood was determined according to the 15-item Geriatric Depression Scale in the Korean version (short form of GDS-K; SGDS-K) [17]. The best cut-off value for screening depressive mood was proposed as ≥8 (total scores ranged from 0 to 15). In our study, scores of 8 or more and less than 8 were classified as “depressed” and “not depressed”, respectively.

### 2.4. Statistical Analysis

**Figure 2:**
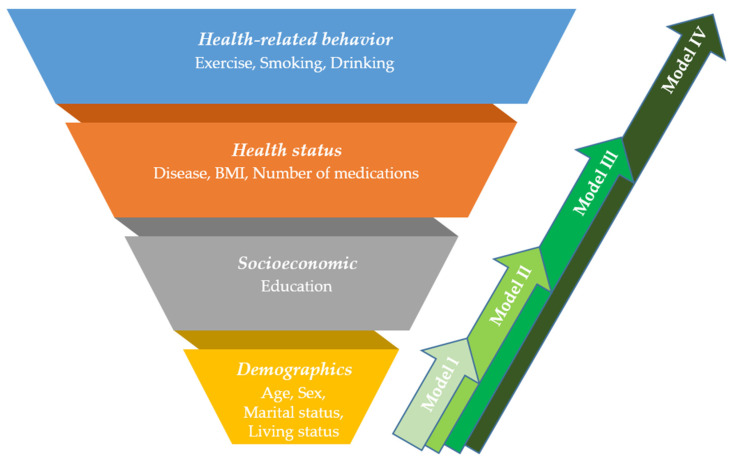
Suggested individual-variable adjusted models in this study. The demographic, socioeconomic, health status, and health-related behavior subcategories were combined, and four models were established.

Descriptive statistics were performed using the χ^2^ or *t*-test to compare differences in the subjective health status in individual, physical, and psychological categories. After analyzing each independent variable for univariate regression, statistically significant variables were chosen to carry out multivariate regression. After that, we built new individual-variable adjusted models to reduce the potential confounding effects of individual variables. Elements from individual categories (including demographic, socioeconomic, health status, and health-related behavior subcategories) were combined into groups and were entered into regression models one by one. In designing the model, we assumed that variables in the demographic subcategory (i.e., age, sex, marital status, and living status) were the least modifiable among the four subcategories. Other subcategories, such as socioeconomic, health status, and health-related behaviors could be more easily modified (depending on one’s own effort) in consecutive order. Based on this hypothesis, we first built Model I (consisting of only the demographic subcategory), and then we encompassed easily modifiable subcategories in a stepwise way, which were presented as Models II, III, and IV (Model I: demographic variables only, Model II: demographic and socioeconomic variables, Model III: demographic, socioeconomic, and health status variables, Model IV: all the individual variables in four subcategories) (Figure 2). Odds ratios (ORs) and corresponding 95% confidence intervals (CIs) were also calculated. The statistical significance referred to a value of *p* < 0.05. Data were analyzed using the IBM SPSS software, version 22.0 (IBM, Armonk, NY, USA).

## 3. Results

### 3.1. Self-Rated Health Status

Among 4350 participants with lower household income, about half of them (47.5%, *n* = 2066) perceived themselves as unhealthy.

### 3.2. Differences in Individual Characteristics between Groups with Good and Poor SRH

Differences in SRH status according to individual variables are summarized in Table 1. In terms of demographic, socioeconomic, health status, and health-related behavior components, almost all the items (except age and living status) revealed statistical significance between good and poor SRH groups.

### 3.3. Differences in Physical and Psychological Factors between Good and Poor SRH Groups

Statistically significant differences were observed between good and poor SRH in view of physical characteristics. Visual or auditory discomforts, ADL limitation, IADL limitation, and poor nutrition were more frequently observed in the poor SRH group. There also was a significant difference between the two groups in terms of psychological factor such as depression (Table 2).

### 3.4. Multivariable Logistic Regression Analysis of Variables Associated with Poor SRH in Low-Income Seniors Using Individual-Variable Adjusted Models

To assess the physical and mental health factors influencing the risk of poor SRH, we introduced the four individual variable-adjusted models as stated above. In Model IV, poor SRH in lower-income adults was significantly correlated with limitation to ADL (odds ratio (OR): 2.91, 95% confidence interval (CI): 2.12–4.01), limitation to IADL (OR: 1.80, 95% CI: 1.52–2.13), malnutrition (OR: 1.76, 95% CI: 1.52–2.04), and depression (OR: 3.65, 95% CI: 3.10–4.31) in multivariate logistic regression analysis (Table 3). In particular, all four factors were consistently significant from our Model I to IV.

## 4. Discussion

### 4.1. Healthcare Issues among Low-Income Adults

Nowadays, one out of five older adults manages their lives on limited earnings, with little room for additional or unexpected health-related expenses. Low-income seniors are more likely to suffer from a chronic illness that requires medical attention than high-income adults [18]. European studies on healthcare inequality according to socioeconomic status suggested that differences in individual income and education level played a role in generating disparities in one’s health level [19,20,21]. As mentioned earlier, an adult with lower income faces each day with a worse physical and mental sense of well-being.

### 4.2. Self-Rated Health Status in Low-Income Adults

Self-assessed health refers to how individuals evaluate their own health status, and it is a simple but strong indicator for one’s general well-being. This measurement integrates various personal, social, functional, and mental conditions for evaluation. Understanding the importance of individual health status is essential with regard to disease prevention and health promotion [22]. A person with poor SRH is likely to be in physical, emotional, or social distress. As a result, poor SRH could serve as a powerful predictor of frequent outpatient visits, long inpatient stays, and eventual mortality in the elderly. For elderly people who already regard themselves as unhealthy, it is very important to early detect and timely support their physical and psychological shortcomings. As you can imagine, people in a better socioeconomic condition are expected to have better self-perceived health status [23]. On the other hand, we easily assume that the elderly people in a worse socioeconomic position are more ready to give negative answers on self-health assessment items. However, we should also keep in mind that not all low-income adults regard themselves as having poor subjective health. In our survey, less than half of the low-income respondents (47.5%, *n* = 2066) perceived themselves as unhealthy. For this reason, we thought it would be worthwhile to explore and identify factors associated with poor self-reported health in low-income adults. We hoped that insights from our study might help people at risk of negative SRH to live better and happier by early detection and management, ultimately reducing social healthcare burdens.

### 4.3. Factors Affecting Subjective Health in Low-Income Elderly People

Many studies proposed that age, gender, education level, household income, along with many other factors, were in close relationship with one’s subjective health [24]. More often than not, outcomes from this straightforward subjective measurement require careful interpretation depending on individualized circumstances or situations. Herein, we focused on the subjective health of community-dwelling and low-income elderly people. With respect to demographic, socioeconomic, health status, and health-related behavior characteristics, all variables except age and living status showed statistical significance between good and poor SRH groups. Likewise, significant differences were observed between the two groups in terms of physical and mental health characteristics such as visual/auditory discomfort, limitation to ADL/IADL, nutrition, and depression. We made a unique effort to identify relevant items affecting self-assessed health status by controlling individual confounders. To set up new risk-adjusted models, individual factors were grouped into four subcategories. Variables from the demographic subcategory (i.e., age, sex, and marital status) were considered as the least modifiable out of four subcategories. In consecutive order, socioeconomic, health status, and health-related behavior variables were considered more easily modifiable than demographic ones. Instead of combining all individual variables at once, we added subcategories one by one to find out which combinations (or models) would be helpful in controlling confounders. In a multiple logistic regression analysis, we identified elements that were coherent throughout all four models. Four factors (ADL limitation, IADL limitation, poor nutrition, and depression) were statistically significant between good and poor self-perceived health groups.

Older adults with ADL and/or IADL limitations are more dependent on daily living activities. They often complain of poor quality of life (QOL), resulting in an increase in healthcare demand [25]. In our study, both ADL and IADL limitations turned out to be significant risk factors for poor SRH. However, we have to keep in mind that there could be possible bidirectional interactions between limited ADL/IADL and poor SRH; limitations to ADL/IADL can be a consequence of poor SRH and vice versa. Malnutrition is also an important issue in view of QOL and even survival. Malnutrition in seniors is common and is easily overlooked or mismanaged by healthcare providers in the real world [26]. Adequate nutritional assessment and intervention are needed to maintain good nutritional health and sense of well-being.

Depression seemed to be the most powerful predictor of negative SRH in our study. According to the 2014 national survey using the Short Form of Geriatric Depression Scale (SGDS), about a third (33.1%) of people aged 65 and over had depressive symptoms [27]. Mental health in old age was influenced by complex causes: death of a spouse, loss of job, decrease in income, chronic illness, and so on. Difficulties in maintaining good relationships with family members and neighbors also caused mental health problems, including depression [28,29]. In a study on geriatric depression with regard to socioeconomic status, depressive mood was frequently observed when the elderly had any of the following episodes: living alone, receiving insufficient medical care, low education level, smoking/drinking, and reduced physical activities [30]. Another study revealed that negative SRH and inability to perform activities of daily living were in direct association with depressive mood [31].

### 4.4. Strengths and Limitations of Our Research

Our study findings showed that three physical health factors (ADL/IADL limitations and poor nutrition) and a mental health factor (depression) were strongly associated with poor SRH in the elderly with lower household income. In fact, the ADL, IADL, and SRH are multidimensional concepts themselves. Individual, physical, and psychological components affect them either positively or negatively. Based on our findings, any limitation to ADL/IADL, poor nutrition, and depression must be detected as early as possible to improve geriatric subjective health assessment. Good SRH makes a person a healthy member of our society. In addition, healthcare managers and public health policymakers may benefit from our study findings that early detection and management of the elderly at risk of poor SRH reduce social health care expenses in the long run.

Four major limitations could be found in our research. First, this analysis had a cross-sectional design, and this made it difficult to find causality. The cause-and-effect relationship between low income and negative SRH could be unclear, which was an inherent limitation of the study design. Second, there may remain recall bias since the main data source was from the interview. Third, education level was the only item in a socioeconomic subcategory. In other words, other social determinants of health [32] (such as family relations, solidarity network, affiliation, proxy services, and so on) need to be considered in future studies. Finally, our major disadvantage originated from using secondary data as a backbone. It may provide unclear answers to the researcher’s research-related questions or does not contain additional information which the researcher would like to investigate. It requires special attention while interpreting our secondary data analyses. Thus, our study findings necessitate extensive validations in future research.

## 5. Conclusions

Our findings suggest that low-income elderly people with limited ADL/IADL, poor nutrition, and depression are at risk of poor SRH. Considering the importance of SRH in these fragile populations, early recognition (i.e., active screening of malnutrition and depression in the healthcare facilities) and timely intervention (i.e., consultation to rehabilitation services and mental health professionals) are very important. Our sincere efforts might help them to live better and happier, ultimately relieving worldwide healthcare burdens.

## Figures and Tables

**Table 1:** Differences in individual variables between groups with good and poor SRH (*n* = 4350).

Individual Variables		Classification	Good SRH	Poor SRH	χ^2^	*p*
			(*n* = 2284)	(*n* = 2066)		
			*n* (%) or	*n* (%) or		
			M ± SD *	M ± SD *		
Demographic	Age		76.1 ± 6.0	77.0 ± 6.1	4.96	0.979
	Sex	Male	817 (35.8)	588 (28.5)	26.51	<0.001
		Female	1467 (64.2)	1478 (71.5)		
	Marital status	Living with spouse	1120 (49.0)	918 (44.4)	9.23	0.002
		Living without spouse	1164 (51.0)	1148 (55.6)		
	Living status	Alone	1072 (46.9)	1042 (50.4)	7.46	0.059
		Living with spouse	1071 (46.9)	884 (42.8)		
		Living with children	98 (4.3)	99 (4.8)		
		Others	43 (1.9)	41 (2.0)		
Socioeconomic	Education	0–6 years	1580 (69.2)	1626 (78.7)	52.10	<0.001
		7–9 years	345 (15.1)	224 (10.8)		
		10–12 years	280 (12.2)	176 (8.5)		
		≥13 years	79 (3.5)	40 (1.9)		
Health status	Disease	Hypertension	1342 (58.9)	1392 (67.4)	34.52	<0.001
		Diabetes	400 (17.5)	616 (29.8)	91.72	<0.001
		Dementia	24 (1.1)	54 (2.6)	15.05	<0.001
		Arthritis	605 (26.5)	955 (46.2)	183.70	<0.001
	BMI **	Underweight (<18.5)	80 (3.5)	125 (6.1)	34.02	<0.001
		Normal (≥18.5, <25)	1636 (71.6)	1337 (64.7)		
		Overweight (≥25)	568 (24.9)	604 (29.2)		
	Number of medication(s)	0	408 (17.9)	88 (4.3)	466.82	<0.001
		1	308 (13.5)	99 (4.8)		
		2	343 (15.0)	149 (7.2)		
		≥3	1225 (53.6)	1730 (83.7)		
Health-related Behavior	Exercise	None	628 (27.5)	899 (43.5)	167.84	<0.001
		<150 min. a week	472 (20.7)	477 (23.1)		
		≥150 min. a week	1184 (51.8)	690 (33.4)		
	Smoking	Never/Past	207 (9.1)	150 (7.3)	4.68	0.031
		Current	2077 (90.9)	1916 (92.7)		
	Drinking	None	1712 (74.9)	1726 (83.5)	55.16	<0.001
		≤1 standard drink/day	212 (9.3)	157 (7.6)		
		>1 standard drink/day	360 (15.8)	183 (8.9)		

* M ± SD, mean ± standard deviation. ** BMI, body mass index.

**Table 2:** Differences in physical and psychological characteristics between good and poor SRH groups (*n* = 4350).

Physical & Psychological Variables		Classification	Good SRH	Poor SRH	χ^2^	*p*
			(*n* = 2284)	(*n* = 2066)		
			*n* (%)	*n* (%)		
Physical	Visual discomfort	No	1447 (63.4)	1081 (52.3)	54.22	<0.001
		Yes	837 (36.6)	985 (47.7)		
	Hearing discomfort	No	1843 (80.7)	1521 (73.6)	30.94	<0.001
		Yes	441 (19.3)	545 (26.4)		
	ADL * limitation	No	2223 (97.3)	1739 (84.2)	231.14	<0.001
		Yes	61 (2.7)	327 (15.8)		
	IADL ** limitation	No	1815 (79.5)	1110 (53.7)	326.25	<0.001
		Yes	469 (20.5)	956 (46.3)		
	Nutrition	Good	1364 (59.7)	662 (32.0)	333.96	<0.001
		Poor	920 (40.3)	1404 (68.0)		
Psychological	Depression	NoYes	1968 (86.2)	1103 (53.4)	561.42	<0.001
			316 (13.8)	963 (46.6)		

* ADL, Activities of daily living. ** IADL, Instrumental activities of daily living.

**Table 3:** Multivariable logistic regression analysis of factors associated with poor SRH in low-income older adults.

Variables	Model I	Model II	Model III	Model IV
	OR (95% CI)	OR (95% CI)	OR (95% CI)	OR (95% CI)
Visual discomfort	1.24 (1.08–1.43) ***	1.23 (1.07–1.42) ***	1.10 (0.92–1.31)	1.09 (0.95–1.27)
Hearing discomfort	1.08 (0.91–1.27)	1.08 (0.91–1.28)	1.14 (0.98–1.31)	1.11 (0.93–1.32)
ADL * limitation	3.24 (2.38–4.41) ***	3.25 (2.38–4.42) ***	3.11 (2.26–4.27) ***	2.91 (2.12–4.01) ***
IADL ** limitation	2.05 (1.75–2.40) ***	1.99 (1.70–2.33) ***	1.91 (1.61–2.25) ***	1.80 (1.52–2.13) ***
Poor nutrition	2.10 (1.82–2.41) ***	2.09 (1.82–2.40) ***	1.73 (1.50–2.00)***	1.76 (1.52–2.04) ***
Depression	3.77 (3.22–4.40) ***	3.73 (3.19–4.37) ***	3.80 (3.23–4.48) ***	3.65 (3.10–4.31) ***

Model I: adjusted for demographic (age, sex, marital status, and living status) subcategory only. Model II: adjusted for demographic and socioeconomic (education) subcategories. Model III: adjusted for demographic, socioeconomic, and health status (disease, BMI, and number of medications) subcategories. Model IV: adjusted for demographic, socioeconomic, health status, and health-related behavior (exercise, smoking, and drinking) subcategories. * ADL, Activities of daily living. ** IADL, Instrumental activities of daily living. *** *p* < 0.05.

## Data Availability

Our data are readily available upon reasonable request.
